# Inflammatory signatures distinguish metabolic health in African American women with obesity

**DOI:** 10.1371/journal.pone.0196755

**Published:** 2018-05-08

**Authors:** Gerald V. Denis, Paola Sebastiani, Kimberly A. Bertrand, Katherine J. Strissel, Anna H. Tran, Jaromir Slama, Nilton D. Medina, Guillaume Andrieu, Julie R. Palmer

**Affiliations:** 1 Cancer Center, Boston University School of Medicine, Boston, Massachusetts, United States of America; 2 Department of Pharmacology and Experimental Therapeutics, Boston University School of Medicine, Boston, Massachusetts, United States of America; 3 Department of Biostatistics, Boston University, Boston, Massachusetts, United States of America; 4 Slone Epidemiology Center, Boston University, Boston, Massachusetts, United States of America; 5 Division of Plastic and Reconstructive Surgery, Boston University School of Medicine, Boston, Massachusetts, United States of America; Dasman Diabetes Institute, KUWAIT

## Abstract

Obesity-driven Type 2 diabetes (T2D) is a systemic inflammatory condition associated with cardiovascular disease. However, plasma cytokines and tissue inflammation that discriminate T2D risk in African American women with obese phenotypes are not well understood. We analyzed 64 circulating cytokines and chemokines in plasma of 120 African American women enrolled in the Black Women’s Health Study. We used regression analysis to identify cytokines and chemokines associated with obesity, co-morbid T2D and hypertension, and compared results to obese women without these co-morbidities, as well as to lean women without the co-morbidities. We then used hierarchical clustering to generate inflammation signatures by combining the effects of identified cytokines and chemokines and summarized the signatures using an inflammation score. The analyses revealed six distinct signatures of sixteen cytokines/chemokines (*P* = 0.05) that differed significantly by prevalence of T2D (*P* = 0.004), obesity (*P* = 0.0231) and overall inflammation score (*P* < E-12). Signatures were validated in two independent cohorts of African American women with obesity: thirty nine subjects with no metabolic complications or with T2D and hypertension; and thirteen breast reduction surgical patients. The signatures in the validation cohorts closely resembled the distributions in the discovery cohort. We find that blood-based cytokine profiles usefully associate inflammation with T2D risks in vulnerable subjects, and should be combined with metabolism and obesity counselling for personalized risk assessment.

## Introduction

Unresolved, low level inflammation is a defining feature of Type 2 diabetes (T2D), in which specific pro-inflammatory cytokines drive disease pathogenesis [1,2]. These mechanisms were first convincingly demonstrated in cellular studies showing that acute exposure of differentiated adipocytes to very low concentrations of tumor necrosis factor (TNF)-α was sufficient over hours to ablate insulin-stimulated glucose uptake [3], providing a functional definition of insulin resistance [4]. Specific local inflammatory events in adipose tissue [5] and other organs, such as the liver, have been intensively studied in obesity (body mass index (BMI) ≥ 30 kg/m^2^) [6]. Among the first histological features of inflamed fat to be identified were CD68+ pro-inflammatory macrophages that abnormally infiltrate adipose tissue depots [7,8] and encircle stressed or apoptotic adipocytes in metabolically abnormal patients [9,10] and animal models [11]. These macrophages produce TNF-α and other cytokines that worsen insulin resistance in the adipose tissue. Cytokines originating in the immune infiltrates and abnormal adipocytes spill out of adipose tissue and other organs into the circulation, rendering the patient systemically inflamed. Plasma signatures of inflammation that mirror these local biomarkers of insulin resistance have also been investigated for many years [12,13]. In addition to TNF-α, cytokines including interleukin (IL)-1β, IL-6 [14]and C-reactive protein (CRP) are systemically elevated in patients with T2D [15] and stratify risks for cardiovascular complications [16,17]. Thus, local and systemic inflammation is informative of the severity of metabolic disease and its complications.

However, clinical studies have shown that different components of metabolic syndrome and different measures of metabolic health associate with different inflammatory signatures [18]. Furthermore, the strength of the association with specific disease risks (cardiovascular disease, T2D, stroke) depends on how metabolic health is defined [19], and which cytokines are analyzed. Most studies have assessed only a limited number of cytokines for disease association. Certain populations that are relatively metabolically healthy despite obesity [20,21] have proven informative in this regard, because in general such patients are protected from obesity-associated co-morbidities [22] and are less inflamed [23] or have elevated cardioprotective cytokines [24]. The healthy obese population is a significant fraction of the U.S. adults with obesity, and depending on how metabolic health is measured, can represent about a quarter of subjects, including among U.S. racial and ethnic minority populations [25].

The purpose of this investigation was to identify new cytokines associated with metabolic disease. We took a more comprehensive and unbiased approach than has been reported previously, and used well validated, multiplex cytokine profiling kits to assay panels of cytokines in the plasma of obese participants in the Black Women’s Health Study, with and without co-morbid T2D and hypertension, compared to subjects who were both lean and metabolically healthy. In view of the barriers to personalized medicine experienced by African American women, and their disproportionately high occurrence of cardiometabolic disease and obesity-associated cancer mortality, intensive investigation of this population for informative blood signatures is urgently required. Simple, peripheral blood-based profiling of cytokines has the potential to provide useful information to understand and better monitor cardiometabolic risks in at-risk and vulnerable subjects.

### Rationale and methodology

Although plasma measurements of single cytokines, such as IL-6 or CRP, have shown significant differences in mean values when two groups of different metabolic health or BMI are compared in large clinical studies, other key cytokines, such as TNF-α, frequently do not show the expected differences [13], which has puzzled investigators. It is possible that individual cytokines fluctuate significantly over time during T2D progression, or rise and fall as secondary changes in response to other cytokines. These features strongly suggest that a systems analysis based on multiple biomarkers will have greater interpretive power. A signature that averages responses across multiple cytokines should reduce the effect of outliers and generate a pattern of cytokines associated with metabolic health. Differences in these patterns as a function of metabolic complications should be clearer and more robust than departures from normal ranges of individual cytokines. We have successfully employed such an agnostic, data-driven clustering methodology to develop signatures of healthy aging using systemic biomarkers in the Long Life Family Study [26]. The approach identified specific signatures that are associated with lower mortality, morbidity and physical function, and were distinct from signatures with higher risks for morbidity and mortality. We considered that this approach might be well suited to cross sectional analysis of cytokines of a different group, participants in the Black Women’s Health Study with different subtypes of obesity defined by the presence or absence of T2D.

## Results

### Cytokine signatures associated with metabolically abnormal obesity

**Fig 1:**
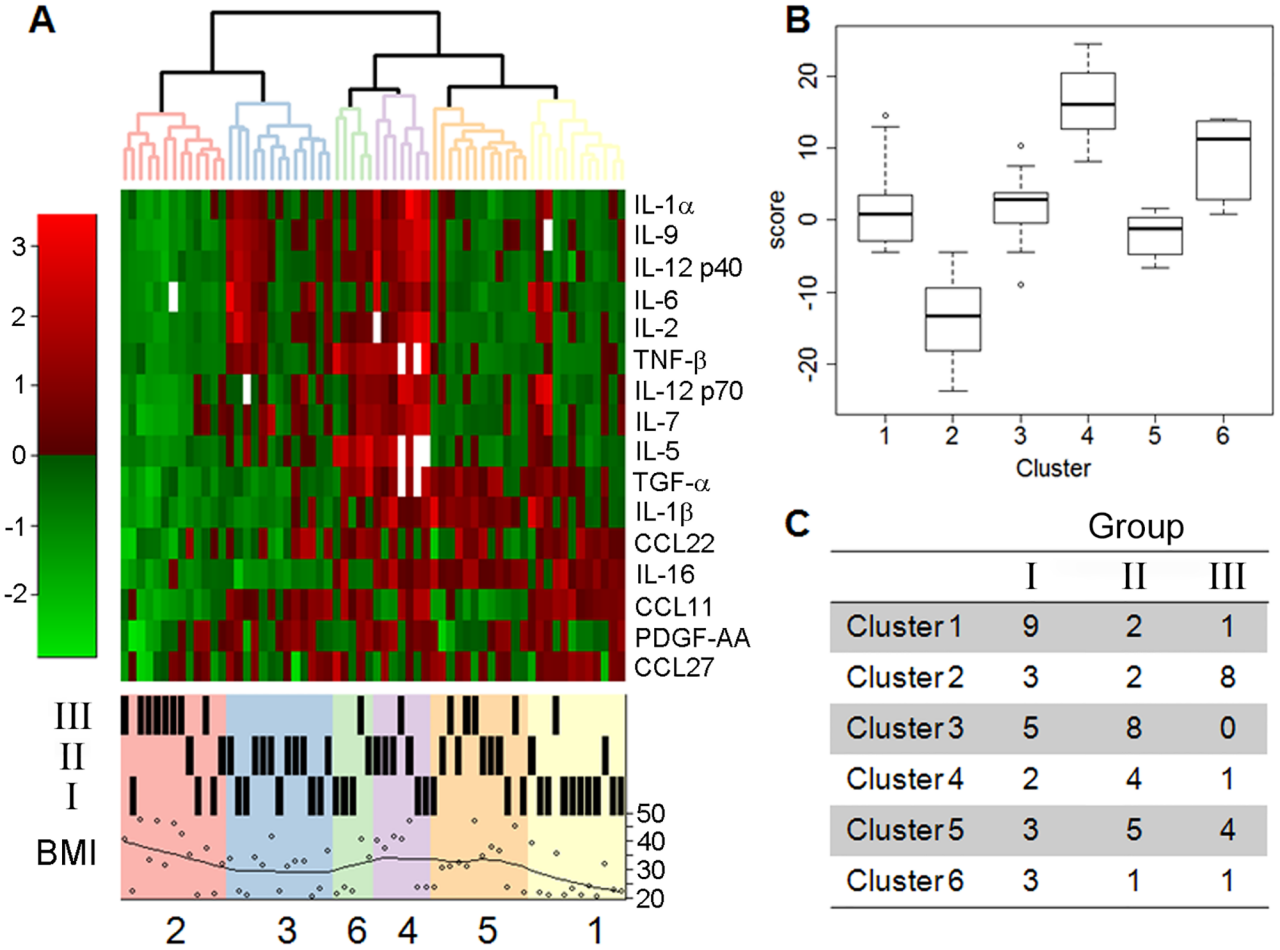
Hierarchical clustering analysis of sixteen signature cytokines/chemokines in BWHS subjects. A) Transformed median fluorescence intensities of cytokines detected by multiplex assay are displayed by row and subjects by column in the heat map. Red indicates increased expression and green decreased expression, according to fold-change scale at left. Clusters were detected by cutting the dendrogram at a height seen in less than 1% of randomly generated dendrograms. B) Six clusters of subjects according to inflammation score. C) Representation of each cluster according to metabolic Group: I, normal BMI without T2D or hypertension (‘lean, healthy’); II, obese without T2D or hypertension (‘obese, healthy’); III, obese with both T2D and hypertension, not treated with metformin (‘obese, unhealthy, no metformin’).

We used plasma samples and clinical and demographic data from 120 participants from the Black Women’s Health Study (BWHS), an ongoing prospective cohort study initiated in 1995 [27]. Subjects were classified as either normal weight (BMI < 24 kg/m^2^) without either T2D or hypertension (‘lean, healthy’: Group I); obese (BMI ≥ 30 kg/m^2^) without either T2D or hypertension (‘obese, healthy’: Group II); obese with both T2D and hypertension, not treated with metformin (‘obese, unhealthy, no metformin’: Group III); and obese with both T2D and hypertension, and treated with metformin (‘obese, unhealthy, metformin-treated’: Group IV) (Table 1). The groups differed in age at blood draw, upper *vs* lower body obesity, metabolic parameters and medications. Preliminary analyses showed that use of metformin and non-steroidal anti-inflammatory medications were significantly associated with cytokine levels and could confound the effects of BMI and T2D. Accordingly, we analyzed cytokine data of a subset of sixty two subjects who were not receiving treatment with metformin or non-steroidal anti-inflammatory medications (Table 2) and identified sixteen cytokines that correlated with increased BMI (Group II, ‘obese, healthy’ subjects *vs* Group I, ‘lean, healthy’ subjects), and with T2D independently of BMI (Group III, ‘obese, unhealthy, no metformin’ subjects *vs* Group II, ‘obese, healthy’ subjects). Table 3 lists the sixteen cytokines. We then used hierarchical clustering of these sixteen cytokines among the sixty two subjects to generate six clusters of subjects with significantly different cytokine signatures (*P* = 0.05), as shown in the heatmap in Fig 1A.

**Table 1:** Characteristics of four groups of BWHS subjects.

	Lean, healthy	Obese, healthy	Obese, unhealthy, no metformin	Obese, unhealthy, metformin-treated	*P-*value
Group	I	II	III	IV	
**N**	30	30	27	33	
**Mean age, y (SD)**	51.40 (4.10)	52.03 (4.86)	53.22 (4.17)	55.06 (3.66)	0.003
**WHR**	0.76 (0.04)	0.81 (0.08)	0.88 (0.07)	0.85 (0.07)	< 0.001
**Median BMI, kg/m**^**2**^ **(range)**	22.07 (19.86–23.94)	33.97 (30.21–46.91)	39.09 (30.89–49.53)	39.32 (30.44–47.16)	< 0.001
**HbA1c, % (SD)**	5.71 (0.43)	5.67 (0.32)	7.31 (1.95)	6.86 (0.96)	< 0.001
**T2D duration, y**			8.33 (5.55)	7.30 (5.59)	> 0.050
**Metformin**	0	0	0	33	
**NSAID**	5	8	12	21	0.0565*

Data are means ± SD for normally distributed variables. *P*-values were computed using ANOVA and χ^2^ test (*).

Abbreviations: BMI, Body Mass Index; HbA1c, glycated hemoglobin; NSAID, non-steroidal anti-inflammatory; T2D, Type 2 diabetes; WHR, waist to hip ratio.

**Table 2:** Characteristics of BWHS subjects after exclusion of subjects taking metformin or non-steroidal anti-inflammatory medications.

	Lean, healthy	Obese, healthy	Obese, unhealthy, no metformin	*P-*value
Group	I	II	III	
**N**	25	22	15	
**Mean age, y (SD)**	51.28 (4.1)	52.32 (4.86)	52.47 (4.17)	>0.05
**WHR**	0.76 (0.04)	0.80 (0.08)	0.87 (0.07)	< 0.001
**Median BMI, kg/m**^**2**^ **(range)**	21.99 (20.10–23.94)	34.21 (30.58–46.91)	40.39 (30.92–47.66)	<0.001
**HbA1c, % (SD)**	5.7 (0.43)	5.67 (0.32)	8.38 (1.95)	<0.001

Abbreviations: BMI, Body Mass Index; HbA1c, glycated hemoglobin; WHR, waist to hip ratio.

**Table 3:** Cytokine correlations in BWHS.

	β I:II	SE I:II	*P-*value	β III:II	SE III:II	*P-*value
**IL-1α**	-0.07	0.14	>0.05	**-0.41**	0.16	**0.012**
**IL-9**	-0.10	0.15	>0.05	**-0.49**	0.17	**0.004**
**IL-12 p40**	-0.18	0.16	>0.05	**-0.46**	0.20	**0.018**
**IL-6**	-0.22	0.18	>0.05	**-0.60**	0.22	**0.006**
**IL-2**	-0.01	0.09	>0.05	**-0.26**	0.10	**0.008**
**TNF-β**	-0.09	0.29	>0.05	**-0.73**	0.34	**0.030**
**IL-12 p70**	-0.06	0.12	>0.05	**-0.32**	0.14	**0.023**
**IL-7**	-0.16	0.10	>0.05	**-0.31**	0.13	**0.013**
**IL-5**	-0.20	0.19	>0.05	**-0.53**	0.22	**0.015**
**TGF-α**	-0.15	0.18	>0.05	**-0.46**	0.22	**0.037**
**IL-1β**	-0.10	0.26	>0.05	**-0.63**	0.30	**0.032**
**CCL-22**	-0.20	0.11	>0.05	**-0.35**	0.12	**0.004**
**CCL-11**	**0.46**	0.22	**0.039**	-0.31	0.29	>0.05
**PDGF-AA**	-0.12	0.17	>0.05	**-0.47**	0.20	**0.017**
**CCL-27**	**0.20**	0.08	**0.022**	-0.01	0.10	>0.05

β I:II = change of log(cytokine) comparing ‘lean, healthy’ group (I) *vs* ‘obese, healthy’ group (II); SE I:II: standard error of β I:II from regression model adjusted for age and white blood cell count.

β III:II = change of log(cytokine) comparing ‘obese, unhealthy, no metformin’ group (III) *vs* ‘obese, healthy’ group (II); SE III:II: standard error of β III:II from regression model adjusted for age and white blood cell count.

Bold font of numerical values identifies the significant comparisons with respect to ‘obese, healthy’ (II) as the reference group.

**Fig 2:**
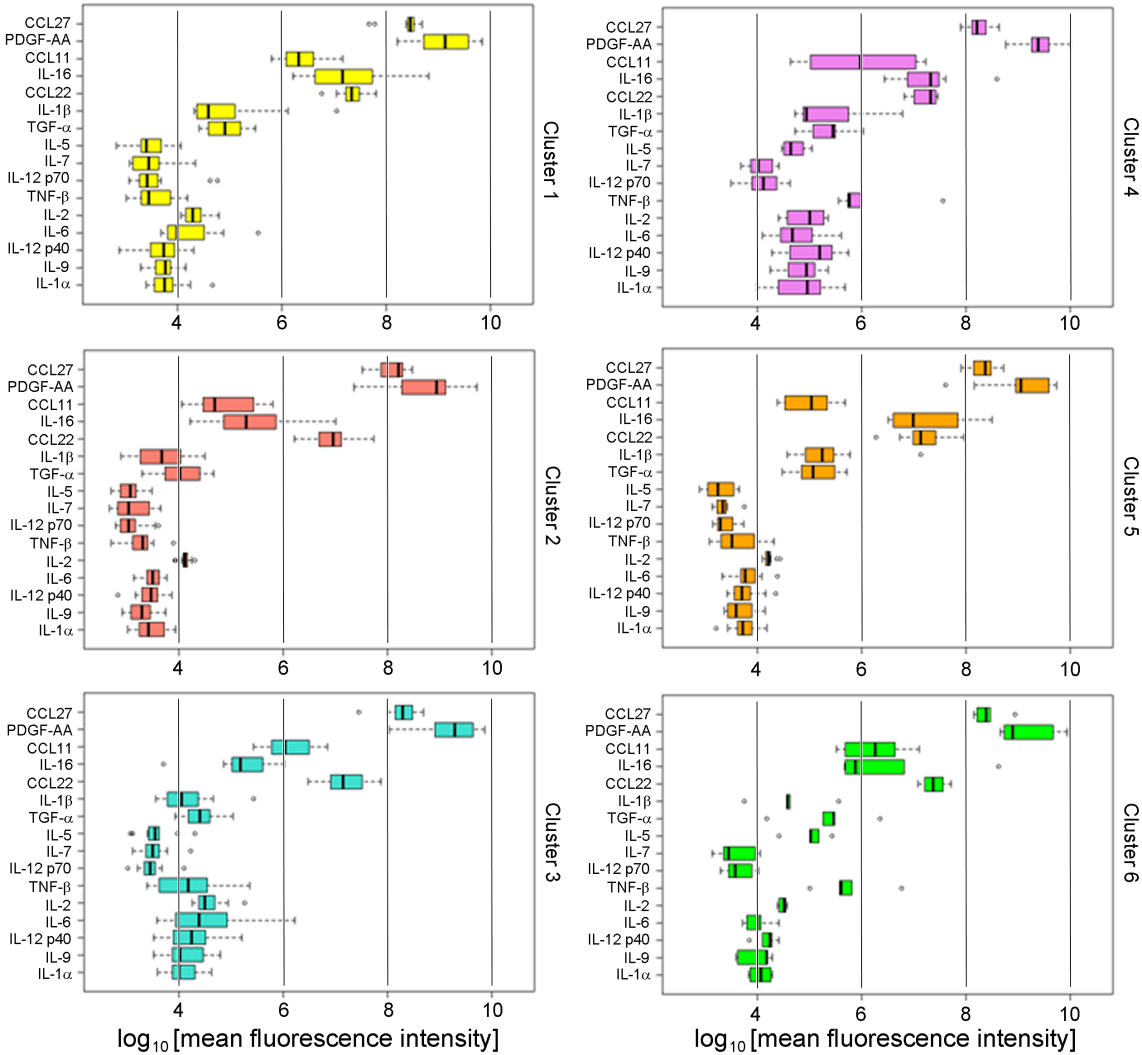
Signature patterns of BWHS subjects. Quantification of sixteen cytokines (median fluorescence intensity in logarithmic scale) that resolve the six clusters of the signature are shown with side-by-side boxplots. Individual cytokines are identified at left: CCL27, C-C motif chemokine ligand 27, also known as CTACK; PDGF-AA, platelet-derived growth factor α polypeptide; CCL11, C-C motif chemokine ligand 11; IL-16, interleukin 16; CCL22, C-C motif chemokine ligand 22; IL-1β, interleukin 1β; TGF-α, transforming growth factor α; IL-5, interleukin 5; IL-7, interleukin 7; IL-12 p70, interleukin 12, 70 kDa subunit; TNF-β, tumor necrosis factor β; IL-2, interleukin 2; IL-6, interleukin 6; IL-12 p40, interleukin 12, 40 kDa subunit; IL-9, interleukin 9; IL-1α, interleukin 1α. Vertical guide bars shown for reference to permit comparison among clusters. Subjects reporting that they were taking metformin or NSAIDs were excluded.

We developed a novel ‘inflammation score’, similar to an ‘aging score’ we previously reported [26] by taking the sum of the sixteen standardized cytokine values for each subject. We then used this score to describe the distribution of the overall levels of inflammation that associate with the six signatures (Fig 1B). The inflammation scores associated with the six signatures were statistically different (Table 4, *P* < 0.001). Interestingly, more than half of the subjects with obesity, T2D and hypertension who were not treated with metformin (Group III) fell into a single cluster (Cluster 2), whereas 75% of subjects in Cluster 1 were lean, normal BMI and no T2D (Group I), and 62% of subjects in Cluster 3 were obese but no T2D (Group II) (Fig 1C and Table 4). The cytokines associated with Clusters 4, 5 and 6 showed an intermediate patterns between Clusters 1, 2 and 3 and comprised a mixture of subjects from Groups I, II and III. The clusters differed significantly by prevalence of T2D (*P* = 0.004), BMI (*P* = 0.023) and the overall inflammation score (*P* < E-12) (Table 4). Each signature is characterized by a distinct pattern of the sixteen cytokines can be clearly represented with side-by-side boxplots (Fig 2), analogous to our previously published approach [26].

**Table 4:** Correlation of signatures and metabolic groups in BWHS subjects without reported use of metformin or non-steroidal anti-inflammatory medications.

	Cluster						
	1	2	3	4	5	6	*P-*value
**Cluster size, N**	12	13	13	7	12	5	
**T2D, N (%)**	1 (8)	8 (62)	0 (0)	1 (14)	4 (33)	1 (20)	**0.004**
**Mean BMI (SD)**	25.23 (6.51)	35.11 (9.55)	29.45 (6.86)	36.20 (9.12)	32.87 (7.95)	28.25 (8.47)	**0.023**
**Mean inflammation score (SD)**	**2.03** (6.14)	**-13.68** (6.19)	**1.57** (5.16)	**16.39** (5.78)	**-2.03** (2.88)	**8.54** (6.22)	**< 0.001**

Correlation of signatures of subjects, *without reported use* of metformin or NSAIDs, who were allocated to the six signatures. BMI mean (row 2) also indicates the percentage of subjects in each cluster with T2D. *P*-values were calculated using χ^2^ tests and ANOVA.

Abbreviations: BMI, Body Mass Index; T2D, Type 2 diabetes.

**Fig 3:**
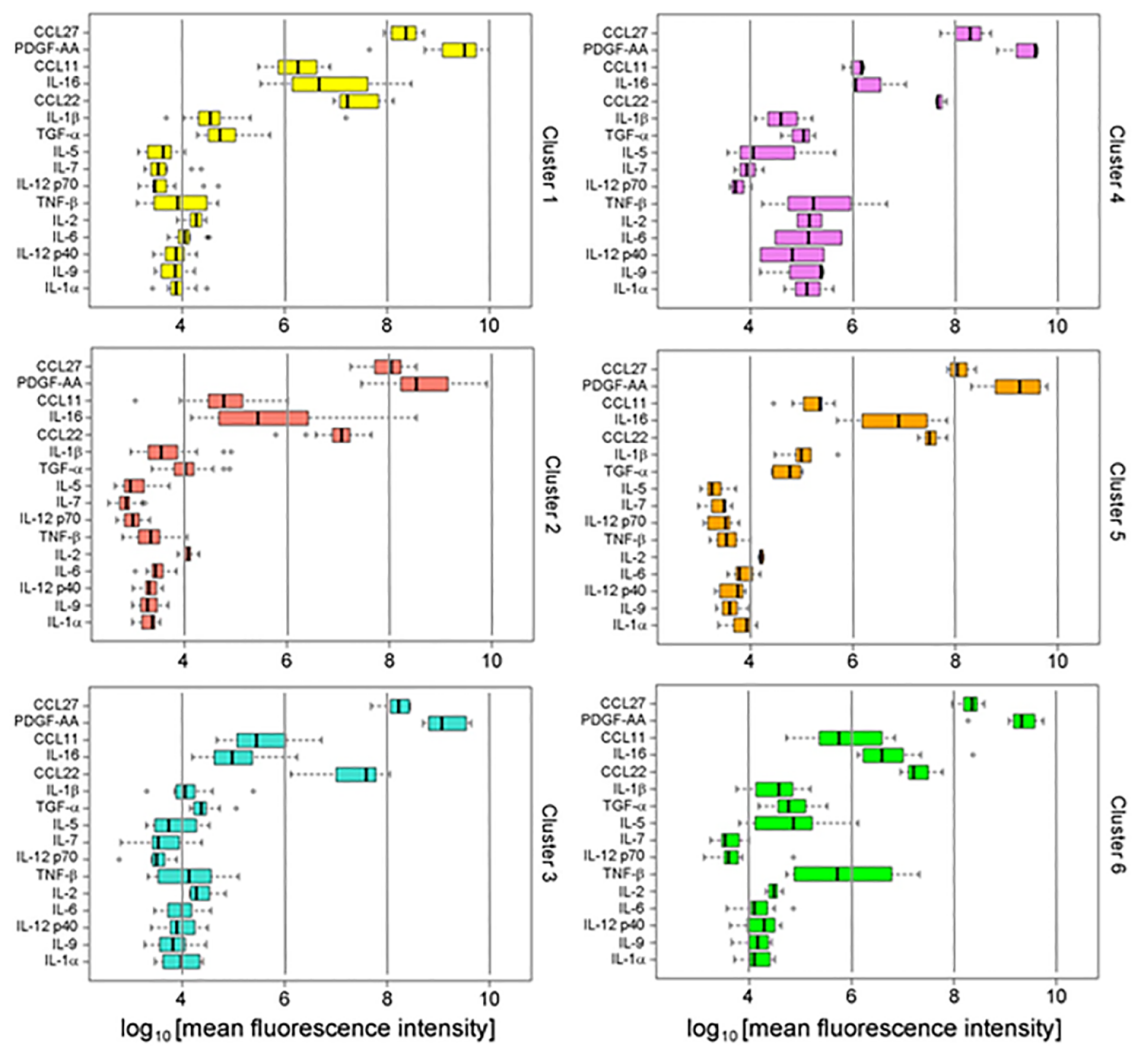
Six-cluster signature in BWHS subjects taking metformin or NSAIDs. Patterns of sixteen cytokines by signatures assigned with the multi-label classification algorithm. The algorithm used the profiles of cytokines discovered in the original BWHS set of sixty two subjects to infer the most likely signature of fifty eight other subjects, who had been excluded from the first analysis because they were taking medications. The six signatures are very similar to those calculated for Fig 2 subjects, who were not taking medications. Only the distribution of CCL22 in Cluster 3 differed significantly (*P*-value = 0.003) from the distribution of CCL22 in patients assigned to the same Cluster 3 in the discovery set (Fig 2). For all other signatures, the difference in distributions of individual cytokines by cluster was tested using t-test and no other comparison reached statistical significance after Bonferroni correction for multiple testing (*P* < 0.003).

To test the effects of metformin and nonsteroidal anti-inflammatory drug (NSAID) treatment on cytokine signatures, we used a multi-label classification algorithm [26] to assign fifty eight subjects, who had been excluded from the initial analysis, to one of the six signatures based on their sixteen cytokine values. The algorithm assigned each subject to one signature with high confidence (median entropy score -0.03). Furthermore, the patterns of cytokines were highly similar to those generated in the first set of subjects, who had not been treated with these medications, suggesting that these cytokine signatures are reproducible and robust (Fig 3). Summary characteristics of these subjects are presented in Table 5. Correlations were determined for signatures and metabolic groups in BWHS subjects, who were taking metformin or anti-inflammatory drugs, and who were individually classified into one of the six clusters of signatures (Table 6). Cluster 2 comprised the largest percentage of T2D subjects (95%) and the smallest percentage of metformin-treated T2D subjects (67%) (Table 6). In contrast, 83% of the twelve subjects assigned to Cluster 1 were T2D but 80% of them were treated with metformin. The distribution of the inflammation score of these fifty eight subjects was similar to the distribution of the inflammation score in the discovery set and differed significantly among the six clusters (*P* < 1E-16). The overall level of inflammation of subjects assigned to Cluster 1 was elevated (mean score 6.12) compared to the level of inflammation of subjects in Cluster 1 of the discovery set (mean score 2.03), but this difference did not reach statistical significance (*P* = 0.11).

**Table 5:** Clinical characteristics and metabolic groups of BWHS subjects with reported use of metformin or non-steroidal anti-inflammatory medications.

	Cluster						
	1	2	3	4	5	6	*P*-value
**Cluster size, N**	12	19	10	3	7	7	
**Group I (lean, healthy), N**	2	1	0	0	1	1	0.086
**Group II (obese, healthy), N**	0	0	2	1	4	1	0.086
**Group III (obese, unhealthy, no metformin), N**	2	6	2	0	1	1	0.086
**Group IV (obese, unhealthy, metformin-treated), N**	8	12	6	2	1	4	0.086
**T2D + HTN, %**	83.	95.	80.	67.	29.	71.	**0.017**
**T2D + metformin, %**	80.	67.	75.	100.	50.	80.	0.092
**NSAID, %**	75.	79.	80	33.	100.	86.	> 0.05
**Group I, %**	17.	5.	0.	0.	14.	14.	0.084
**Group II, %**	0.	0.	20.	33.	57.	14.	
**Group III, %**	17.	32.	20.	0.	14.	14.	
**Group IV, %**	67.	63.	60.	67.	14.	57.	
**Smoking, % (current)**	8.	0.	0.	0.	14.	0.	> 0.05
**Smoking, % (never)**	50.	63.	80.	100.	57.	86.	
**BMI mean (2013) kg/m**^**2**^	35.44	37.33	35.99	35.11	36.14	34.95	> 0.05
**WHR (2013)**	0.85	0.84	0.85	0.86	0.85	0.88	> 0.05
**SES (2013)**	0.06	0.02	-0.37	0.04	-0.37	-0.24	> 0.05
**HbA1c (2013), %**	7.27	6.63	7.15	5.67	6.24	6.47	> 0.05
**Duration T2D, years**	5.60	9.00	9.50	6.00	6.50	7.60	> 0.05
**ΔBMI (2013–2011)**	1.44	-0.45	-0.11	1.11	-0.72	-1.05	> 0.05
**Inflammation score**	**82.05**	**70.60**	**79.06**	**85.00**	**77.47**	**84.58**	**2.08E-13**

Characteristics of BWHS subjects, *with reported use* of metformin or NSAIDs, who were allocated to the six signatures. *P*-values were calculated using χ^2^ tests and ANOVA.

Abbreviations: BMI, Body Mass Index; HbA1c, glycated hemoglobin; HTN, hypertension (systolic BP = 140–159 mmHg, diastolic BP > 85 mmHg); NSAID, non-steroidal anti-inflammatory drug; SES, socioeconomic score; T2D, Type 2 diabetes; WHR, waist to hip ratio.

**Table 6:** Correlation of signatures and metabolic groups in BWHS subjects with reported use of metformin or anti-inflammatory drugs.

	Cluster						
	1	2	3	4	5	6	*P-*value
**Cluster size, N**	12	19	10	3	7	7	
**T2D, N (%)**	10 (83)	18 (95)	8 (80)	2 (67)	2 (29)	5 (71)	**0.017**
**T2D on metformin, N (%)**	8 (80)	12 (67)	6 (75)	2 (100)	1 (50)	4 (80)	> 0.05
**NSAID users, N (%)**	9 (75)	15 (79)	8 (80)	1 (33)	7 (100)	6 (86)	> 0.05
**Mean BMI, kg/m**^**2**^ **(SD)**	35.44 (8.26)	37.33 (6.41)	35.99 (4.31)	35.11 (2.11)	36.14 (8.38)	34.95 (8.06)	> 0.05
**Mean inflammation score (SD)**	**6.12** (6.04)	**-12.39** (4.62)	**2.11** (5.11)	**10.21** (4.37)	**0.58** (3.05)	**11.31** (4.63)	**< 1E-16**

Correlation of signatures of subjects, *with reported use* of metformin or NSAIDs, who were allocated to the six signatures. Rows 2–4 show the percentages of subjects in each cluster. Percentage with T2D (row 2), percentage of T2D subjects who reported use of metformin (row 3) and the percentage of subjects who reported use of anti-inflammatory medications (row 4). *P*-values were calculated using χ^2^ tests and ANOVA.

Abbreviations: BMI, Body Mass Index; NSAID, non-steroidal anti-inflammatory drug; T2D, Type 2 diabetes.

### Validation of clusters in independent cohorts

**Fig 4:**
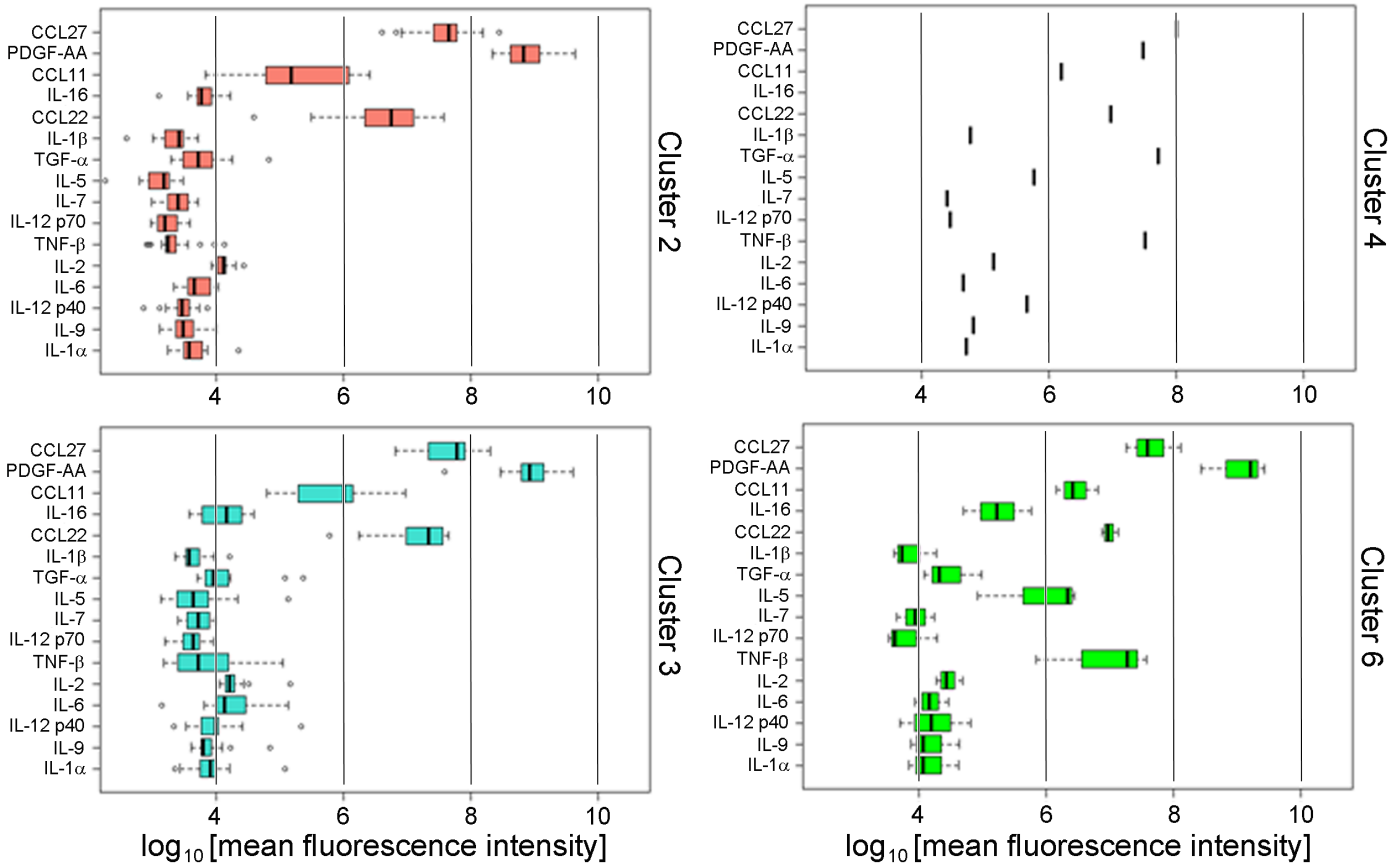
Cluster signature in Komen subjects for validation of BWHS–derived signature. The patterns of cytokines in the four clusters matched very closely the patterns discovered in the original BWHS data set, although CCL27 and IL-16 in Cluster 2 and Cluster 3 were significantly lower compared to the discovery set (*P* < 0.0002).

Although the cluster signature appeared to be robust and reproducible within the BWHS cohort, it was important to test the validity of the signature in other stand-alone cohorts, who had been recruited through unrelated methodology, but share African American self-reported race and obesity. We therefore tested the cluster signature in an independent group of thirty nine African American women with obesity and either no metabolic complications, or who had both T2D and hypertension, who had voluntarily contributed blood samples to the Susan G. Komen Tissue Bank. Subject characteristics are reported in Table 7. The multi-label classification algorithm was used to assign each of the thirty nine subjects to one of the cytokine signatures discovered in the sixty two BWHS samples. No examples of Cluster 1 or 5 were found in this analysis, which was expected because no lean women without metabolic complications were included in this cohort. The thirty nine Komen subjects were assigned to Clusters 2, 3, 4 and 6 with high certainty (median entropy score -0.001), and the distributions of cytokines stratified by clusters (Fig 4) closely resembled the distributions discovered in BWHS subjects (Fig 2). The classification of subjects based on their cytokine signatures worked well to identify subjects with T2D (Table 8): 73% of subjects assigned to Cluster 2 were T2D, and only 50% of T2D subjects were not treated with metformin. Subjects assigned to Cluster 3 had on average the greatest BMI (37.61 kg/m^2^; SD 8.85). Subjects in Cluster 2 had higher levels of inflammation (mean score -6.51) compared to subjects in Cluster 2 of the discovery set (mean score -13.68) and the difference was significant (*P* = 0.00173).

**Table 7:** Characteristics of African American female volunteers with obesity, who participate in the Susan G. Komen Tissue Bank.

	ND	T2D	*P*-value
**N**	11	28	
**Mean age, y (SD)**^a^	57.1 (7.2)	58.9 (7.7)	> 0.05
**Mean BMI, kg/m**^**2**^ **(SD)**^a^	30.4 (4.0)	36.5 (7.4)	**0.002**
**Medication**^b^			
** Metformin (%)**	0	12 (43)	**0.048**
** Oral anti-inflammatory (%)**	5 (45)	8 (29)	> 0.05
** Anti-hypertensive (%)**	4 (36)	22 (79)	> 0.05
** Lipid-lowering (%)**	2 (18)	16 (57)	> 0.05
** Hypertension (%)**	6 (54)	21 (75)	> 0.05

^a^ p-value (*P*) computed from t-test.

^b^ p-value from Fisher exact test

Clinical and demographic characteristics of subjects have been previously reported [50].

**Table 8:** Correlation of signatures and metabolic groups in Komen subjects with reported use of metformin or anti-inflammatory drugs.

	Cluster				
	2	3	4	6	*P-*value
**Cluster size, N**	22	13	1	3	
**T2D, N (%)**	16 (73)	11 (85)	0	1 (33)	> 0.05
**T2D drug, N (%)**	7 (44)	8 (73)	--	0 (0)	1
**T2D using metformin, N (%)**	8 (50)	4 (36)	--	0 (0)	1
**NSAID users, N (%)**	7 (32)	5 (38)	0	1 (33)	> 0.05
**HTN, N (%)**	16 (73)	10 (77)	0	1 (33)	> 0.05
**HTN drug, N (%)**	6 (27)	3 (23)	1 (100)	3 (100)	**0.021**
**CVD, N (%)**	3 (14)	3 (23)	0	2 (67)	> 0.05
**CVD and HTN drug, N (%)**	6 (27)	2 (15)	0	0	> 0.05
**Lipid lowering drug, N (%)**	11 (50)	7 (54)	0	0	> 0.05
**Mean BMI, kg/m**^**2**^ **(SD)**	33.72 (5.85)	37.61 (8.85)	39.45	28.49 (0.49)	> 0.05
**Mean age, y (SD)**	59.41 (7.20)	57.92 (8.39)	62.00	51.67 (5.03)	> 0.05
**Mean inflammation score (SD)**	**-6.51** (4.72)	**4.57** (6.37)	**31.40**	**17.46** (10.16)	**< 0.001**

Characteristics of Komen subjects [50] allocated to four of the six signatures discovered in the BHWS. T2D (%), percentage of subjects in each cluster with T2D; T2D drug, percentage of T2D subjects using T2D drug; T2D using metformin, percentage of subjects using metformin. *P*-values were calculated using χ^2^ tests and ANOVA.

Abbreviations: BMI, Body Mass Index; CVD, cardiovascular disease; HTN, hypertension; NSAID, non-steroidal anti-inflammatory drug; T2D, Type 2 diabetes.

**Fig 5:**
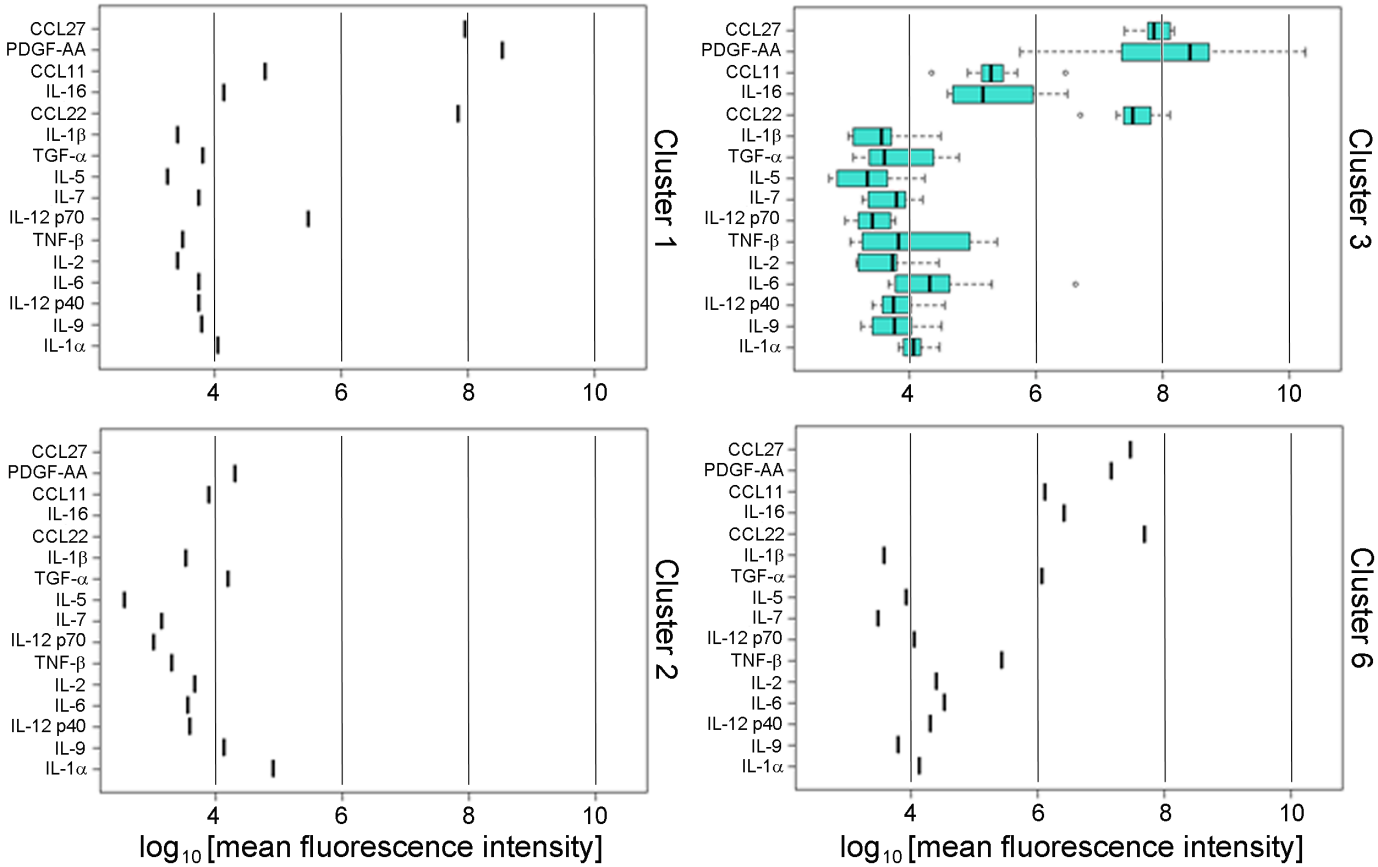
Cluster signature in breast reduction surgical subjects for validation of BWHS–derived signature. The four patterns of clustered cytokines found in the surgical subjects with obesity were very similar to the patterns identified in the original BWHS data set (Fig 2) [28], as well as the independent cohort of Komen subjects (Fig 4) [50].

For further independent validation of the signature, we profiled an independent cohort of thirteen African American women with obesity who were pre-menopausal, and who had presented for elective breast reduction surgery at Boston Medical Center in the Department of Plastic Surgery, as we have previously described [28]. One subject was diagnosed with T2D, whereas the others had neither T2D nor uncontrolled hypertension. Subjects with pre-diabetes, based on HbA1c > 5.7%, were excluded (Table 9), and plasma was profiled in the same manner as for all other cohorts. The cluster signature prediction was performed again with high confidence (median entropy score -0.0001), and ten subjects were assigned to Cluster 3, while the remaining three subjects were assigned to Clusters 1, 2, and 6. No subjects with Cluster 4 or 5 were found in this cohort (Fig 5). The clustering method correctly assigned the single subject with T2D into Cluster 2 (Table 10). The inflammation score of the ten subjects assigned to Cluster 3 (mean score -0.15) was lower than the inflammation score of subjects of the discovery set in Cluster 3 (mean score 1.57), but the difference was not statistically significant (*P* = 0.58).

**Table 9:** Characteristics of African American female surgical patients with obesity, who presented at Boston Medical Center for elective breast reduction.

	ND	T2D
**N**	12	1
**Age, y**		
Mean (SD)	41.1 (13.60)	56
Median (range)	44.5 (19–58)	-
**BMI, kg/m**^**2**^		
Mean (SD)	34.35 (2.95)	35.20
Median (range)	35.20 (27.4–37.90)	-
**Diabetes measures**^a^		
HbA1c, % (SD)	5.50 (0.58)	7.80
Fasting glucose, mg/dL (SD)	94.36 (8.78)	141.00
Fasting insulin, μU/mL (SD)	11.10 (6.49)	6.00
HOMA-IR (SD)	2.40 (1.50)	2.09
**Blood analytes**^b^		
Cholesterol, mg/dL (SD)	177.10 (16.21)	137.00
HDL cholesterol, mg/dL (SD)	48.82 (12.43)	27.00
LDL cholesterol, mg/dL (SD)	110.00 (24.08)	93.00
Triglycerides, mg/dL (SD)	91.45 (28.49)	85.00
Adiponectin, total, ng/mL (SD)	3098.5 (823.5)	ND
Adiponectin, HMW, ng/mL (SD)	609.1 (334.7)	ND
Estradiol, pg/mL (SD)	46.10 (32.18)	16.00
FSH, mIu/mL (SD)	17.04 (16.96)	52.00
LH, mIu/mL (SD)	8.64 (6.28)	19.10
**Medication**		
Metformin	0	0
Anti-inflammatory (oral)	8	1
Anti-hypertensive	5	0
Lipid-lowering	3	1
**Medical history**		
T2D	0	1
Hypertension^c^	5	0
Asthma	1	0
Stroke	0	0
Arthritis	1	0
Thyroid	1	1
Osteoporosis	0	0
Renal	0	0
Anxiety/depression	4	0

^a^ Three subjects were classified as pre-diabetic, based on HbA1c > 5.7%, and were excluded from the non-diabetic group.

^b^ Menopausal status was determined by estradiol, FSH and LH, and self-report.

^c^ Well controlled hypertension was not excluded.

Abbreviations: BMI, Body Mass Index; FSH, follicle-stimulating hormone; HbA1c, glycated hemoglobin; HDL, high density lipoprotein; HMW, high molecular weight; HOMA-IR, homeostatic model assessment for insulin resistance; LDL, low density lipoprotein; LH, luteinizing hormone; ND, non-diabetic; T2D, Type 2 diabetes.

**Table 10:** Correlation of signatures and metabolic groups in Boston Medical Center surgical cohort.

	Cluster				
	1	2	3	6	*P-*value
**Cluster size, N**	1	1	10	1	
**T2D, N (%)**	0	1 (100)	0	0	--
**T2D drug, N (%)**	0	1 (100)	0	0	--
**T2D on met-formin, N (%)**	0	1 (100)	0	0	--
**NSAID users, N (%)**	1 (100)	1 (100)	6 (60)	1 (100)	1
**HTN, N (%)**	0	0	4 (40)	1 (100)	> 0.05
**HTN drug, N (%)**	1 (100)	1 (100)	6 (60)	0	> 0.05
**CVD, N (%)**	1 (100)	1 (100)	9 (90)	1 (100)	1
**CVD and HTN drug, N (%)**	0	0	1 (10)	0	1
**Lipid lowering drug, N (%)**	0	1 (100)	1 (10)	0	> 0.05
**Mean BMI, kg/m**^**2**^ **(SD)**	31	35.64	34.34 (2.97)	37.54	> 0.05
**Mean age, y (SD)**	28	56	34.8 (12.53)	58	> 0.05
**Mean inflammation score (SD)**	**-0.95**	**-7.11**	**-0.15** (8.39)	**9.52**	**> 0.05**

Characteristics of BMC surgical patients allocated to four of the six signatures. T2D (%), percentage of subjects in each cluster with T2D; T2D drug, percentage of T2D patients on T2D drug; T2D on metformin, percentage of subjects treated with metformin; NSAID, non-steroid anti-inflammatory drugs; CVD, cardiovascular disease; HTN, hypertension. *P*-values were calculated using χ^2^ tests and ANOVA.

Abbreviations: BMI, Body Mass Index; CVD, cardiovascular disease; HTN, hypertension; NSAID, non-steroidal anti-inflammatory drug; T2D, Type 2 diabetes.

## Discussion

Nearly one in five deaths in adults (age 40–85 years) in the U.S. can be attributed to obesity [29]. Furthermore, African American adults bear a greater burden of obesity-associated mortality than Whites [30], including cancer mortality. African American women with obesity (BMI ≥ 30 kg/m^2^) experience elevated risks for cardiovascular disease and T2D, compared to U.S. Whites [31]. Among all U.S. racial and ethnic categories, elevated BMI is a leading cause of preventable morbidity, a major risk factor for cardiovascular disease and all-cause mortality in cardiovascular disease [32], for T2D [33,34] and for several cancers [35].

Although obesity and metabolically abnormal, inflamed adipose tissue are jointly associated with risks of cardiometabolic disease and cancer, there is also a long acknowledged subset of obese adults who display relatively normal metabolic and inflammatory profiles, who have been termed ‘metabolically healthy’ obese [6,20,21,23–25]. The reduced inflammation associated with this metabolically healthy obese subgroup has been associated with reduced risk for cardiovascular complications of obesity [17,18,22]. Metabolic inflammation likely positively associates with risk for certain obesity-driven cancers, such as breast cancer in post-menopausal women and colon cancer [36–38]. Understanding the biologic differences between individuals with metabolically healthy and unhealthy obesity could aid development of novel tissue and plasma-based biomarkers, such as cytokines and chemokines, which are more informative of cardiometabolic and cancer risks than is obesity alone. Here, we focused on obese African American women because of the great public health need for noninvasive, precision medicine profiles to benefit this high-risk group. Our analysis revealed six signatures of sixteen cytokines that correlate with obesity, with and without T2D and hypertension. The analysis in three independent cohorts showed that these signatures are common.

In designing the cross sectional analysis, we had hypothesized that Group III subjects (with obesity, T2D and hypertension, who were unmedicated with metformin) would have the highest inflammation. In a longitudinal study with follow-up, these subjects would be predicted to experience the greatest risk for cardiovascular complications, stroke and obesity-driven cancers, compared to subjects who are lean, healthy and lacking in metabolic disease (Group I). We further hypothesized that Group II subjects (obese, yet lacking T2D and hypertension) should have intermediate levels of inflammation. Multiplex analysis of plasma cytokines might associate with metabolic differences among the three groups. Novel biomarkers could help uncouple disease-associated inflammation from body phenotype, and plasma could assist non-invasive monitoring of women who have different cardiometabolic risks. During the initial analysis, we learned that metformin treatment of obese and metabolically unhealthy subjects significantly reduces expression of inflammatory cytokines in plasma, thus it was necessary to resolve two groups of obese and unhealthy subjects, into one treated with metformin (Group IV) and the other without this treatment (Group III). This result also validates independent studies showing that metformin reduces inflammation in metabolic disease. Unbiased analysis of differentially expressed cytokines revealed a relatively small set of analytes (S1 Fig), which when considered together in an inflammation score, provide great statistical power to resolve the groups. Based on this unbiased analysis, we were able to conclude that some metabolically healthy subjects with obesity have a similar pattern of inflammation as lean and healthy subjects, associated with Cluster 3. Nevertheless, there were some subjects that would be expected to be healthy and uninflamed by routine clinical examination, who share patterns of elevated inflammation more typical of metabolic disease. Thus, we suggest that blood based monitoring of specific cytokines should accompany usual clinical measures like BMI, in order to provide the most informative profile of patient risk. Longitudinal studies of this population will be necessary to determine the predictive value of the clusters for specific disease incidence.

Although most adults with obesity are inflamed, paradoxically, long duration obesity has also been associated with impaired immune responses, including deficient B cell antibody production in response to immunization [39–41], and likely weakened tumor surveillance through depleted CD8+ T cells [42]. The unresolved inflammation of chronic metabolic disease has been considered to weaken normal immune responses, leading to an ‘immune exhausted’ state that is more vulnerable to infections and cancer. In recent work, we have shown in BWHS subjects that T2D of duration ≥ 5 years is associated with increased incidence of estrogen-receptor negative breast cancer, compared to subjects without T2D [43], suggesting failure of immune surveillance and elevated metastasis risk [44]. The crosstalk between human metabolic regulation and both innate and adaptive immune systems is not well understood, but is central to mechanisms that link obesity to cardiometabolic and cancer risks, and has prompted major effort to translate insights from obese animal models into clinically relevant biomarkers associated with specific disease risks.

We observed that Cluster 2, with a relatively low inflammation score, contained the highest fraction of women with obesity and co-morbid T2D and hypertension. Our interpretation of this signature is that subjects with long duration T2D have developed an immune exhaustion phenotype, and are developing immunological defects reported for reduced immunization responses and *ex vivo* T cell function. Specifically, the T2D subjects in Cluster 2 had an average 7.75 years since the T2D was first diagnosed compared to an average of 3.75 years among subjects in Cluster 5, although the difference was not significant (*P*-value 0.16). Interestingly, Cluster 1 subjects, who have elevated BMI, also have a higher inflammation score, suggesting that metabolic disease and obesity each contribute independently to overall levels of inflammation.

Subjects with obesity but without a diagnosis of diabetes had the highest inflammation score (Cluster 4), consistent with the possibility that subjects in this group are more likely to progress to T2D diabetes. Certain cytokines in the clusters have already been established as distinguishing among high inflammatory, metabolically abnormal and low inflammatory, metabolically healthy subtypes of obesity, such as IL-6 [18,45]. CCL11/Eotaxin, a plasma factor associated with asthma in obesity, has been positively associated with metabolically abnormal human obesity and declines after weight loss [46]. Other cytokines in the clusters have not previously been associated with T2D or human obesity, such as CCL27/CTACK. Reduced PDGF-ββ has been associated with the overall reduced inflammatory profile of lean and healthy humans, but does not resolve metabolically unhealthy obese from metabolically healthy obese humans [45]. Interestingly, both IL-1α and IL-1β are reduced in Cluster 2 subjects, similar to gene expression patterns reported for innate immune challenge of peripheral blood lymphocytes of aged adults (≥ 65 years) compared to younger adults (< 40 years) [47], supporting interpretations that metabolically unhealthy obesity resembles some inflammatory features of aging [39–41].

### Strengths and limitations

We conducted the most comprehensive survey yet of plasma cytokines and chemokines, in order to identify biomarkers of ‘metabolically unhealthy’ obesity in African American women, a vulnerable group of adults at high risk for cardiometabolic disease, obesity-driven breast cancer and poor outcomes. Previously, chronic, unresolved inflammation in obesity has been linked to local and systemic production of pro-inflammatory cytokines, particularly C-reactive protein, IL-6, TNF-α, as well as chemotactic and adipokine signaling molecules. The study of obese subjects with and without T2D has demonstrated the utility of blood biomarkers for stratifying risk. However, even as the cardiometabolic and cancer risks of obesity are well appreciated, new work is focusing attention on overweight or normal weight women with abnormal metabolism and elevated inflammatory profiles [48], who may experience elevated risk for certain obesity-driven malignancies like breast cancer [49]. Even as we identified a novel group of cytokines and chemokines that independently associate with T2D risk, 48 of the other analytes tested were not significantly correlated with metabolic disease, which simplifies the task of developing personalized medicine profiles for high risk patients. The set of biomarkers described here can now be examined for potential utility as a prognostic tool and for development of drug targets. Special pairwise cluster comparisons could be attempted if the numbers of subjects in each cluster are sufficient for statistical significance; power was limited for this type of analysis here. We are unable to comment on the utility of these biomarkers for White, Asian or Hispanic women, or to evaluate risks for progression of T2D or cancer, due to the cross-sectional study design. Finally, we were unable to obtain measures of body composition with dexa scan, which would be useful in future to quantify adipose depots and correlate with cytokine and metabolic data.

## Conclusions

BMI alone is a misleading measure of obesity-associated disease risks, and should be combined with profiling of inflammatory cytokines and metabolism to create more personalized risk assessment for African American women with obesity. Interventions and recommendations that are tailored to patients’ inflammatory blood profile could be an important tool for risk assessment of obese individuals, but this step awaits additional, longitudinal investigation.

## Materials and methods

### Subjects

#### Ethics statement

This study and all procedures were approved by the Institutional Review Board of the Boston University Medical Campus, Boston, MA. Informed consent was obtained in writing from each participant at recruitment into the BWHS study, from donors to the Komen Tissue Bank and from surgical subjects before the time of their surgery. The investigation was conducted in accordance with the principles expressed in the Declaration of Helsinki.

#### Discovery cohort: Black Women’s Health Study participants

A total of 59,001 self-identified Black women aged 21–69 years were enrolled in the study in 1995 by completing a questionnaire mailed to subscribers of Essence magazine, as well as to members of the Black Nurses’ Association. The study was approved by the Institutional Review Board of Boston University, Boston, MA. At baseline, information about demographic factors, reproductive history, health history, anthropometric measurements, dietary intake, and use of tobacco, alcohol, vitamins, and select medications was collected via self-administered mailed questionnaires. Participants complete follow-up questionnaires every two years to update outcome, exposure and covariate data; follow-up of the baseline cohort has been complete for 88% of potential years of follow-up through 2013. Clinical and demographic characteristics of the BWHS subjects are reported in Table 5. As part of the overall BWHS, participants were invited to provide a blood sample. Blood collection occurred during 2014 through 2017, with ~13,000 participants providing a sample by visiting a local Quest Patient Service Center for the blood draw. Cellular components of blood collected in EDTA-treated tubes were removed by centrifugation to generate plasma, which was then aliquoted, frozen for transport on dry ice to Boston University Medical Campus, and maintained at -80°C until thawed for assay of cytokines.

#### Study design

Subjects were assigned to one of four groups as described above. Subject identifiers, questionnaire responses and analytes determined by biochemical assays were linked for analyses.

#### Exposures

Adult height was reported on the baseline questionnaire. Current weight, physician-diagnosed diabetes, and physician-diagnosed hypertension were reported at baseline and on each follow-up questionnaire. BMI was defined as weight in kilograms divided by height in meters squared. Women who reported diabetes diagnosed after 30 years of age were considered to have T2D. Women who reported hypertension and treatment with hypertension medications or diuretics were considered to have hypertension, as in our previous BWHS analyses. Values for BMI, T2D and hypertension were taken from the 2013 questionnaire, which was completed < 12 months before the blood draw.

#### Validation cohort 1: Komen volunteers

Plasma was collected from thirty nine female donors through the Susan G. Komen for the Cure^®^ Tissue Bank, at the Simon Cancer Center (Indiana University), frozen and transported on dry ice to Boston University as above. All subjects self-identified as African American and were sorted into two groups with BMI > 25 kg/m^2^. One group (N = 11) was non-diabetic but obese, the other had T2D and obesity (N = 28) as previously reported [50]. Health histories were obtained, including medications for T2D (metformin), hypertension, hypercholesterolemia or inflammatory conditions that required NSAIDs, and co-morbidities. Clinical and demographic characteristics of the Komen subjects are reported in Table 7. The Komen Tissue Bank samples were collected with approval of the Institutional Review Board of Indiana University.

#### Validation cohort 2: Plastic surgery patients

Self-identified African American women with BMI > 30 kg/m^2^ were recruited during their initial consultation for elective reduction mammaplasty in the Division of Plastic and Reconstructive Surgery clinic at Boston Medical Center. This validation cohort consisted of sixteen women without cancer diagnosis; all were pre-menopausal confirmed by blood hormones (age range 19–51, except one age 58 years). Yes/No questionnaire-reported health history was collected, including age, BMI, T2D, hypertension, hypercholesterolemia, arthritis, osteoporosis, dermatological, renal, respiratory, thyroid, or heart disease and medications. Upon informed consent, blood was drawn by antecubital venous puncture, then glucose, insulin, fatty acids, triglycerides, estradiol, progesterone, high density/low density lipoprotein were determined by clinical assay. Plasma was prepared from EDTA anticoagulated blood by centrifugation, then snap-frozen and maintained at -80°C until assay. Aliquots for cytokine measurements were not subjected to more than two freeze thaw cycles. All subjects ceased taking any NSAIDs ≥ 2 weeks before surgery and were overnight fasted at time of blood draw. Homeostatic Model Assessment of Insulin Resistance (HOMA-IR) was calculated from fasting measures collected just before surgery.

#### Validation cohort 2 exclusions

If surgical subjects had acute infectious illness or fever ≤ 2 weeks before surgery or other chronic or acute inflammatory condition, including autoimmune disease, cancer, poorly controlled hypertension (BP > 160/100), atherosclerotic CVD, stroke, blood disorders or other serious illness, they were excluded. Subjects with Type 1 diabetes were excluded. We determined that three subjects had HbA1c values consistent with pre-diabetes (> 5.7%, < 6.5%) and they were excluded. One subject had HbA1c = 7.8%, consistent with T2D. The remainder of the subjects had HbA1c values in the normal range (< 5.7%, > 4.0%). Thus, we defined one subject as T2D and twelve subjects as neither diabetic nor pre-diabetic, to yield a final cohort of thirteen subjects. Clinical and demographic characteristics of the surgical subjects are reported in Table 9. The Boston University Medical Center Institutional Review Board approved procedures in accordance with the Declaration of Helsinki.

#### Assay of plasma cytokines

We used multiplex kits (EMD Millipore) that permitted antibody capture of plasma analytes in 23-plex format (HCP2MAG-62K-PX23) and 41-plex format (HCYTMAG60PMX41BK) that relies on a magnetic bead system for purification. Beads were handled with a miniaturization drop array and washer system (Curiox Biosystems, Inc.) and signals were quantified by a Luminex Magpix analyzer. Cytokine standards were determined according to the manufacturer’s instructions and curves constructed with xPONENT 4.2 software; instrument calibration and quality control were conducted for each day’s run. Mean Fluorescence Intensities for each analyte were recorded in independent duplicates obtained from a single biological sample and used for statistical analyses.

#### Analysis

Median fluorescence intensity of sixty four cytokines were analyzed as follows. Outliers were removed, as defined by absolute difference from the mean greater than three standard deviations. Data were log-transformed and replicated samples were analyzed with regression models for repeated measures to account for within- and between-subject variability. The models were adjusted by BMI, age and medications as specified. Plate to plate variation was coded as a random effect in the models. Selected markers were used to generate initial signatures, then duplicates were averaged to conduct the cluster analysis. Hierarchical clustering with complete linkage was used to derive clusters and the number of six significant clusters was derived using resampling [51]. See Fig 3 and S2 Fig for details.

The inflammation score of each subject was computed as the sum of standardized cytokines. To infer the cytokine signatures in the three independent datasets, a multi-label Bayesian classifier was trained in the discovery set as described [26]. The classifier computes the probability that each subject in a new data set has signatures of Clusters 1–6, and assigns the signature with maximum posterior probability. To measure the “classification confidence” the entropy of the posterior distribution of cluster assignment was computed as $\sum_{i=1}^{6}p_{i}log(p_{i})$ and the closer to 0, the highest the classification confidence. The minimum classification confidence is log(1/6) = -1.79 that corresponds to a random classification.

## Supporting information

S1 Fig Initial analysis of all sixty-four cytokines. Additional statistical methods and clustering of cytokine signatures from subjects who were treated with metformin or nonsteroidal anti-inflammatory medications.
(DOCX)

S2 Fig QQ plot. (DOCX)
