# Supplementary material for: Inflammatory signatures distinguish metabolic health in African American women with obesity
Source: PLoS One. 2018 May 8;13(5):e0196755. doi: 10.1371/journal.pone.0196755 (PMC5940209; doi:10.1371/journal.pone.0196755)
Supplement: S1 Fig — Additional statistical methods and clustering of cytokine signatures from subjects who were treated with metformin or nonsteroidal anti-inflammatory medications. (DOCX) [file pone.0196755.s001.docx]

**Supporting Information**

Inflammatory signatures distinguish metabolic health in African American women with obesity

Gerald V. Denis^1,2*^, Paola Sebastiani^3^, Kimberly A. Bertrand^4^, Katherine J. Strissel^1^, Anna H. Tran^1^, Jaromir Slama^5^, Nilton D. Medina^5^, Guillaume Andrieu^1^, Julie R. Palmer^4^

^1^ Cancer Center, Boston University School of Medicine, Boston, Massachusetts, United States of America

^2^ Department of Pharmacology and Experimental Therapeutics, Boston University School of Medicine, Boston, Massachusetts, United States of America

^3^ Department of Biostatistics, Boston University, Boston, Massachusetts, United States of America

^4^ Slone Epidemiology Center, Boston University, Boston, Massachusetts, United States of America

^5^ Division of Plastic and Reconstructive Surgery, Boston University School of Medicine, Boston, Massachusetts, United States of America

**Supplementary Figures**

**S1 Fig.
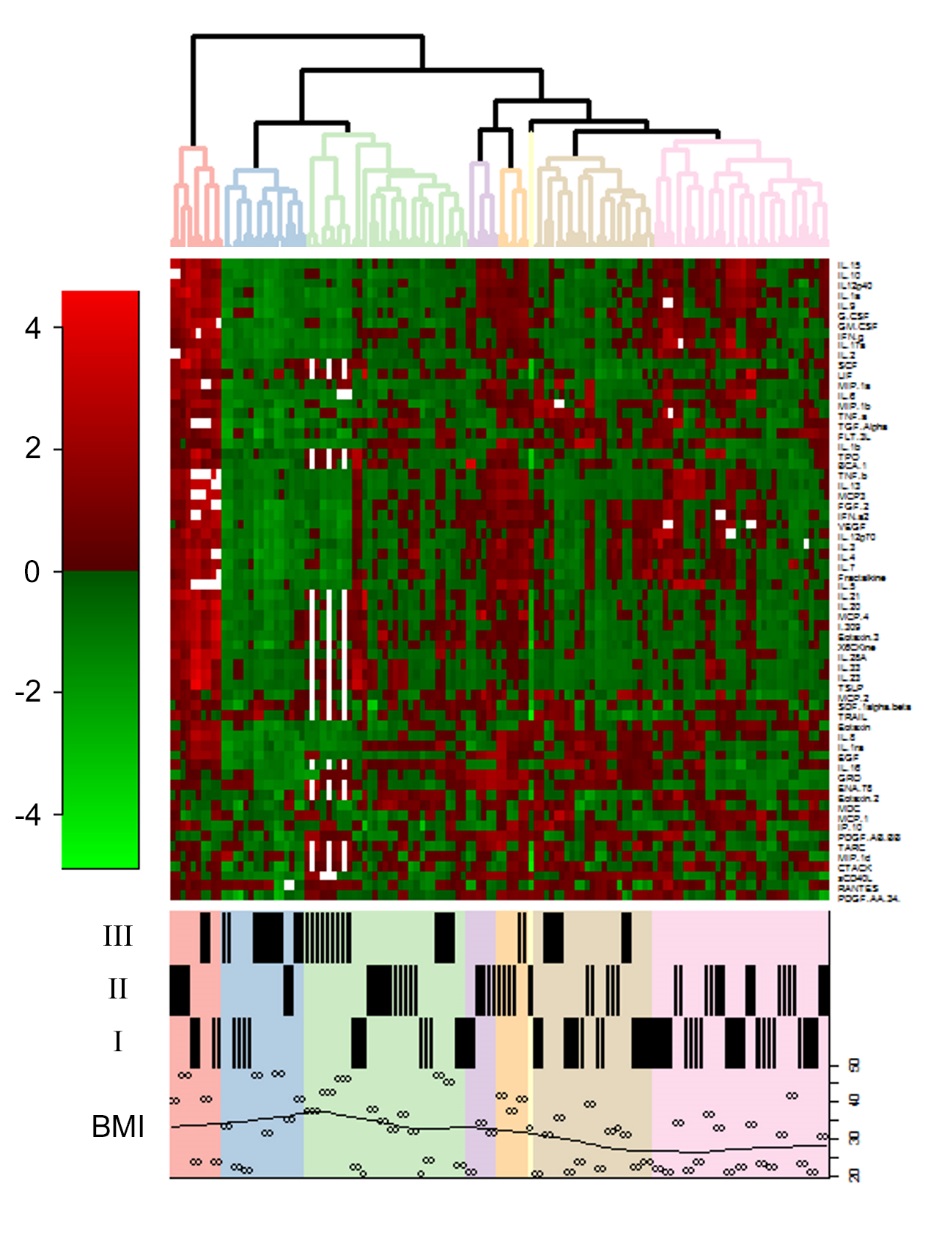
**

**Initial analysis of all sixty-four cytokines.** In the initial analysis, we generated a heat map that combined all cytokines and chemokines, rather than the subset selected by their association with the metabolic groups. Outliers were removed (absolute difference from the mean > 3 standard deviation), then the data were log-transformed. Replicated samples were analyzed with regression models for repeated measures to account for within-subjects variability and between subject variability, and plates were coded as random effects in the models. Then, models were adjusted by age and white blood cell count, whereupon duplicates were averaged and selected markers were used to generate some signatures. There are eight clusters that correlate with T2D but not with obesity. Groups I, II and III are as reported for Figure 1. The analysis showed that clustering of subgroups of subjects is driven by a subset of cytokines, but there is also significant noise in the data. Once we reduced the set of cytokines to the seventeen that were queried in the subsequent analysis, the subgroups were much more clearly resolved.
