# Supplementary material for: Inflammatory signatures distinguish metabolic health in African American women with obesity
Source: PLoS One. 2018 May 8;13(5):e0196755. doi: 10.1371/journal.pone.0196755 (PMC5940209; doi:10.1371/journal.pone.0196755)
Supplement: S2 Fig — (DOCX) [file pone.0196755.s002.docx]

**Supporting Information**

Inflammatory signatures distinguish metabolic health in African American women with obesity

Gerald V. Denis^1,2*^, Paola Sebastiani^3^, Kimberly A. Bertrand^4^, Katherine J. Strissel^1^, Anna H. Tran^1^, Jaromir Slama^5^, Nilton D. Medina^5^, Guillaume Andrieu^1^, Julie R. Palmer^4^

^1^ Cancer Center, Boston University School of Medicine, Boston, Massachusetts, United States of America

^2^ Department of Pharmacology and Experimental Therapeutics, Boston University School of Medicine, Boston, Massachusetts, United States of America

^3^ Department of Biostatistics, Boston University, Boston, Massachusetts, United States of America

^4^ Slone Epidemiology Center, Boston University, Boston, Massachusetts, United States of America

^5^ Division of Plastic and Reconstructive Surgery, Boston University School of Medicine, Boston, Massachusetts, United States of America

**Supplementary Figures**

**S2 Fig.**


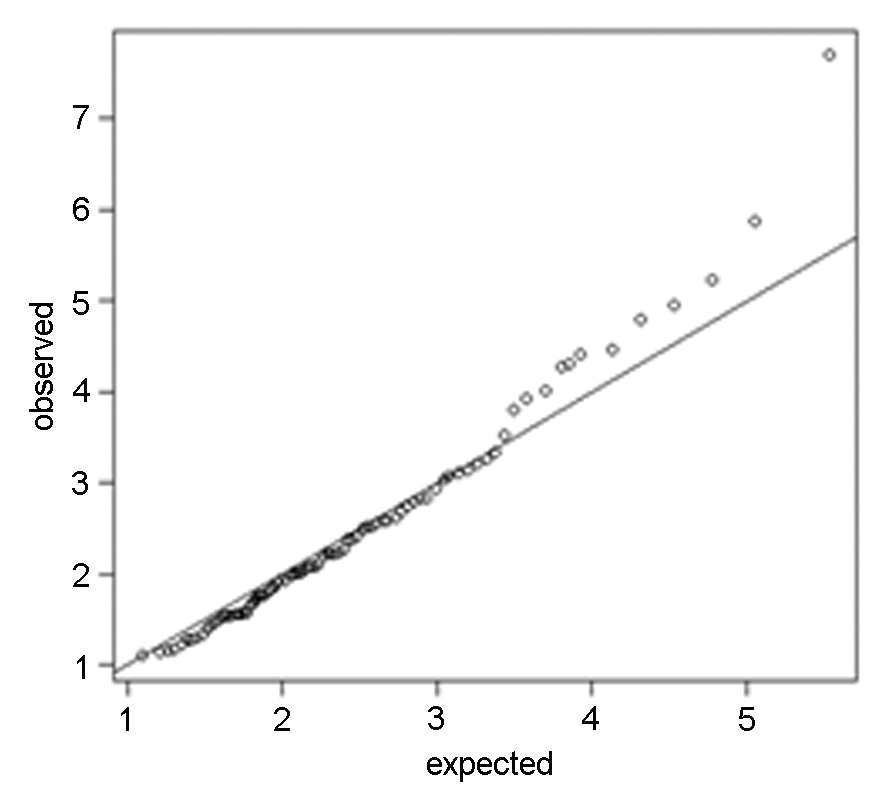


**QQ plot.** The QQ-plot of observed versus expected distance from hierarchical clustering of cytokine profiles was used to infer the number of significant clusters from hierarchical clustering. Briefly, to compute the expected distance under the null hypothesis of no clusters, the data set of 62 subjects and 16 cytokines was reshuffled 10 times to remove biological signals, and the reshuffled data were clustered using hierarchical clustering to generate the reference distance. The departure from the diagonal line suggests the presence of significant clusters and 6 clusters were detected by cutting the observed dendrogram at the 95^th^ percentile of the reference distance.
